# Supplementary material for: A Humanized Yeast Phenomic Model of Deoxycytidine Kinase to Predict Genetic Buffering of Nucleoside Analog Cytotoxicity
Source: Genes (Basel). 2019 Sep 30;10(10):770. doi: 10.3390/genes10100770 (PMC6826991; doi:10.3390/genes10100770)
Supplement: Supplementary file 1 [file genes-10-00770-s001.zip › Additional_Files/Additional_File7_GOTermHeatmaps.zip!/Additional_File7_GOTermHeatmaps/ribosome_assembly.pdf]

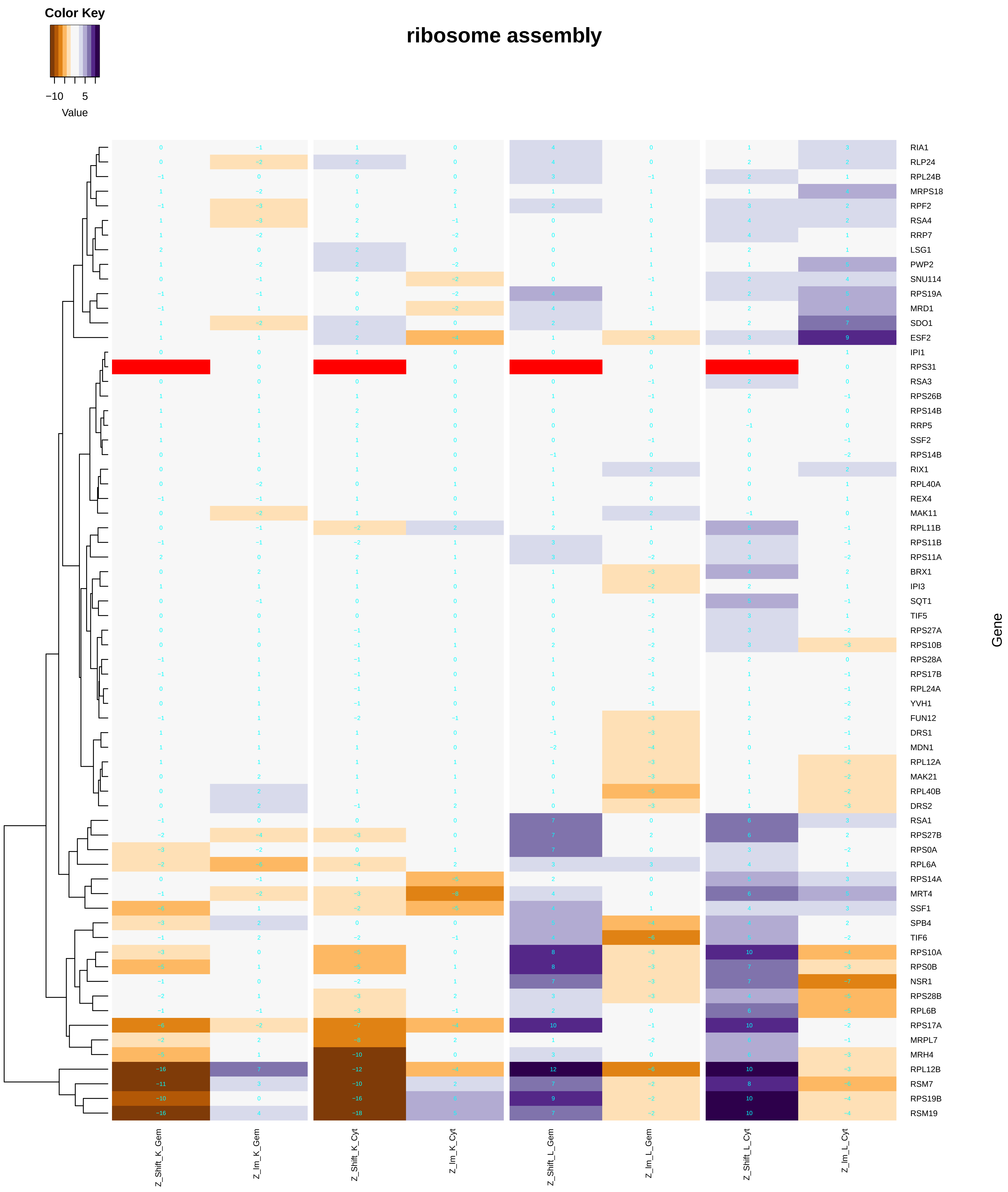

Color Key
−10 5
Value
ribosome assembly
RIA1
RLP24
RPL24B
MRPS18
RPF2
RSA4
RRP7
LSG1
PWP2
SNU114
RPS19A
MRD1
SDO1
ESF2
IPI1
RPS31
RSA3
RPS26B
RPS14B
RRP5
SSF2
RPS14B
RIX1
RPL40A
REX4
MAK11
RPL11B
RPS11B
RPS11A
BRX1
IPI3
SQT1
TIF5
RPS27A
RPS10B
RPS28A
RPS17B
RPL24A
YVH1
FUN12
DRS1
MDN1
RPL12A
MAK21
RPL40B
DRS2
RSA1
RPS27B
RPS0A
RPL6A
RPS14A
MRT4
SSF1
SPB4
TIF6
RPS10A
RPS0B
NSR1
RPS28B
RPL6B
RPS17A
MRPL7
MRH4
RPL12B
RSM7
RPS19B
RSM19
Gene
Z_Shift_K_Gem
Z_Im_K_Gem
Z_Shift_K_Cyt
Z_Im_K_Cyt
Z_Shift_L_Gem
Z_Im_L_Gem
Z_Shift_L_Cyt
Z_Im_L_Cyt

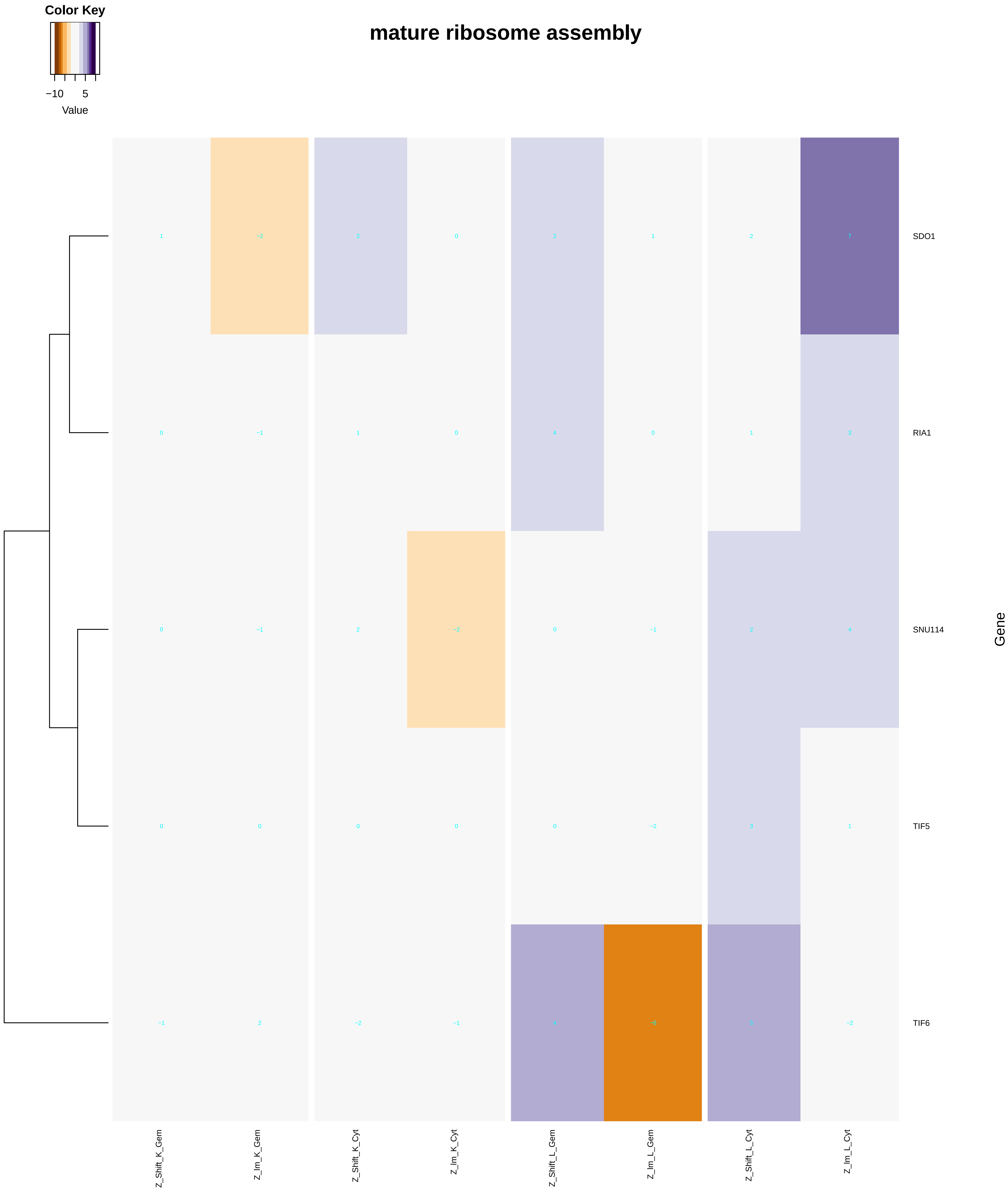

Color Key
−10
5
Value
mature ribosome assembly
SDO1
RIA1
SNU114
TIF5
TIF6
Gene
Z_Shift_K_Gem
Z_Im_K_Gem
Z_Shift_K_Cyt
Z_Im_K_Cyt
Z_Shift_L_Gem
Z_Im_L_Gem
Z_Shift_L_Cyt
Z_Im_L_Cyt
